# Mammalian Cell-Line-Expressed CD2v Protein of African Swine Fever Virus Provides Partial Protection against the HLJ/18 Strain in the Early Infection Stage

**DOI:** 10.3390/v15071467

**Published:** 2023-06-28

**Authors:** Rong-Hong Hua, Jing Liu, Shu-Jian Zhang, Ren-Qiang Liu, Xian-Feng Zhang, Xi-Jun He, Dong-Ming Zhao, Zhi-Gao Bu

**Affiliations:** State Key Laboratory for Animal Disease Control and Prevention, Harbin Veterinary Research Institute, Chinese Academy of Agricultural Sciences, Harbin 150001, China

**Keywords:** African swine fever, African swine fever virus, CD2v protein, p30 protein, K205R protein, subunit vaccine

## Abstract

A cell line expressing the CD2v protein of ASFV was generated. The efficient expression of CD2v protein was determined by immunofluorescence and Western blotting. The CD2v protein was Ni-affinity purified from the supernatant of cell cultures. The CD2v-expressing cells showed properties of hemadsorption, and the secreted CD2v protein exhibited hemagglutinating activity. The antigenicity and immunoprotection ability of CD2v were evaluated by immunizing pigs alone, combined with a cell-line-expressed p30 protein or triple combined with p30 and K205R protein. Immunized pigs were challenged with the highly virulent ASFV strain HLJ/18. Virus challenge results showed that CD2v immunization alone could provide partial protection at the early infection stage. Protein p30 did not show synergistic protection effects in immunization combined with CD2v. Interestingly, immunization with the triple combination of CD2V, p30 and K205R reversed the protection effect. The viremia onset time was delayed, and one pig out of three recovered after the challenge. The pig recovered from ASFV clinical symptoms, the rectal temperature returned to normal levels and the viremia was cleared. The mechanism of this protection effect warrants further investigation.

## 1. Introduction

African swine fever (ASF) is a highly contagious hemorrhagic swine disease with high morbidity and mortality that affects domestic and wild pigs of all ages. Due to its ability to spread rapidly and cause severe illness, ASF has been listed as a notifiable disease by the World Organization for Animal Health (WOAH). The causative agent of ASF is the African swine fever virus (ASFV), which belongs to the *Asfivirus* genus and the *Asfaviridae* family. ASF was first described in Kenya in 1921 and spread throughout Sub-Saharan Africa [1]. In 2007, ASFV was first introduced into Georgia and then spread to other Caucasian and European countries. In 2018, ASFV emerged in China and subsequently spread to other Asian countries [2,3,4]. There are 24 genotypes of ASFV based on the major capsid protein p72 [5,6,7], but the currently circulating virus in Europe, Russia and China has been identified mainly as a highly virulent genotype Ⅱ strain [8,9,10]. The outbreak and epidemic of ASF have caused huge economic losses in the global swine industry.

The linear, double-stranded DNA genome is about 170 kB to 190 kB in size. The virus genome contains more than 150 open reading frames (ORFs) [11]. Although the function of many proteins is unknown, some proteins have been identified that may induce immune protection. Such proteins as p72, p54 and p30 could induce neutralizing antibodies and provide partial protection against virulent ASFV challenges [12,13]. Virus adsorption can be prevented by antibodies to p72 and p54, and virus endocytosis can be preserved by antibodies to p30 [12,14]. Another report showed that a sHA/p54/p30 fusion construct delivered by a baculovirus-based vector provided protection to pigs against ASFV challenge [15]. The surface glycosylated membrane protein CD2v encoded by EP402R mainly involved in hemadsorption is important for protection against homologous ASFV infection [16]. Pigs immunized with baculovirus-expressed CD2v protein developed temporary infection-inhibition antibodies and were protected against lethal infection [17]. Previous studies have demonstrated that K205R is a serological immunodeterminant of ASFV. K205R is recognized by the serum of infected domestic pigs and the antiserum from immunized animals [18,19,20]. The K205R protein is immunogenic; both prokaryotically and eukaryotically expressed K205R induce a strong antibody response in immunized animals [21,22]. However, the role of K205R in the immune protection of ASFV warrants further investigation.

Compared with live attenuated vaccines, subunit vaccines have priority safety. Current ASFV subunit vaccine research focuses mainly on delivering protective antigens and antigen discovery within the ASFV genome. It is known that different ASFV strains have different characteristics, so whether CD2v could provide immune protection against genotype II ASFV currently prevalent in China and other Asian regions must be tested by research. Therefore, in the present study, we constructed several recombinant cell lines to evaluate the immunogenicity of the CD2v, p30 and K205R proteins and immune protection against ASFV strain HLJ/18.

## 2. Materials and Methods

### 2.1. Ethics Statement

Care of laboratory animals and animal experimentation were performed in accordance with animal ethics guidelines and approved protocols. All animal experiments were approved by the Animal Ethics Committee of Harbin Veterinary Research Institute of the Chinese Academy of Agricultural Sciences.

### 2.2. Biosafety Statement and Facility

All experiments with live ASF viruses were conducted within biosafety level 3 (P3) facilities in the HVRI of the CAAS approved by the Ministry of Agriculture and Rural Affairs and China National Accreditation Service for Conformity Assessment.

### 2.3. Cells and Viruses

Baby hamster kidney cells (BHK-21; American Type Culture Collection CCL-10) were cultured at 37 °C in a 5% CO_2_ atmosphere in Dulbecco’s modified Eagle’s medium (DMEM; Gibco, Invitrogen, Carlsbad, CA, USA) supplemented with 10% fetal bovine serum (FBS; Gibco, Grand Island, NY, USA), 100 U/mL penicillin and 100 µg/mL streptomycin (Gibco, Grand Island, NY, USA). Primary porcine alveolar macrophages (PAMs) were collected from 20–30-day-old specific-pathogen-free pigs, and the cells were maintained in RPMI medium (Thermo Scientific, Beijing, China) supplemented with 10% FBS at 37 °C in a 5% CO_2_ atmosphere. Peripheral blood mononuclear cells (PBMCs) were prepared from EDTA-treated swine blood by using a pig PBMC isolation kit (TBD Sciences, Tianjing, China). ASFV strain Pig/Heilongjinag/2018 (HLJ/18) was prepared from the defibrinated blood of virus-inoculated SPF pigs and titrated in PAMs.

### 2.4. Construction of Plasmids 

Genetic codon-optimized cDNAs encoding the tissue plasminogen activator (tPA) signal peptide at amino-terminal, 6 × His tag at carboxy-terminal, fusion with 16–206 of CD2v protein (QBH90546.1), 1–194 of p30 protein (QBH90581.1) and 1–205 of K205R protein (QBH90536.1) in the middle were synthesized. The synthesized cDNAs were cloned into the *Sac*I and *Xho*I sites of the expression vector pCAGneo [23] to generate pCAG-opti-CD2v, pCAG-opti-p30 and pCAG-opti-K205R. The plasmid pCAGneo contains the neomycin resistance gene, which confers resistance to G418. The resulting plasmids were linearized with *Ssp*I and used to transfect BHK-21 cells to construct stable expression cell lines.

### 2.5. Establishment of Stable Cell Lines Secreted Expression of CD2v, p30 and K205R Proteins

Stable cell lines expressing CD2v protein were constructed and selected as previously described [23,24]. Briefly, confluent monolayers of BHK-21 cells were transfected with linearized plasmids using FuGENE HD transfection reagent (Roche Diagnostic GmbH, Mannheim, Germany). Forty-eight hours after transfection, cells were trypsinized and cloned into 96-well plates with medium containing G418. The cloned cells were selected by indirect immunofluorescence assay (IFA) with His-tag-protein-specific mouse monoclonal antibody. The relative expression levels of specific antigen in culture supernatants of cell clones were examined and compared by Western blotting. One clone that showed relatively high expression of His-tagged protein was selected and maintained for further characterization and antigen production. Protein p30 was expressed and purified as previously described [25]. The K205R protein was expressed and purified as previously described [26].

### 2.6. Indirect Immunofluorescence Assays

Preconfluent monolayers were rinsed with phosphate-buffered saline (PBS), fixed with 4% paraformaldehyde at room temperature for 20 min, washed three times with PBS and rinsed for 5 min after each wash, permeabilized with 0.1% Triton X-100 in phosphate-buffered saline (PBS-T) at 4 °C for 10 min, washed with PBS for 5 min at room temperature, blocked with PBS-B solution (PBS containing 4% BSA) at 37 °C for 30 min and incubated with His-tag-specific antibody at 37 °C for 1 h, followed by three washes with PBS. Then, the cells were incubated with fluorescein conjugated goat anti-mouse antibody at room temperature for 1 h, followed by 1 μg/mL 4′,6-diamidino-2-phenylindole dihydrochloride (DAPI) solution incubation for 15 min. Cells were then washed three times as described above and observed using a fluorescence microscope (IMT2 Olympus, Tokyo, Japan).

### 2.7. SDS-PAGE and Western Blotting Analysis

Culture supernatants were concentrated by centrifugation with centrifugal filter unit Vivaspin 500 (GE, 28-9322-25). The samples were mixed with a one-fourth volume of 5 × SDS sample loading buffer with β-mercaptoethanol and boiled for 10 min. Protein samples were separated by 4–20% SDS-PAGE. The gels were stained with QuickBlue protein staining solution (Biodragon, BF06152, Suzhou, China). For Western blot analysis, separated proteins were transferred onto a nitrocellulose membrane and blocked with 5% skimmed milk at 4 °C overnight. Then, the membranes were probed with His-tag-specific antibody at 37 °C for 1 h, washed three times with PBST and incubated with anti-mouse Alexa Fluor 680-conjugated secondary antibodies (Invitrogen, Carlsbad, CA, USA) for 1 h at 37 °C, followed by three washes with PBST. Protein bands were detected with the Li-Cor Odyssey system (Li-Cor Biosciences, Lincoln, NE, USA) and quantified using the Odyssey infrared imaging software (Li-Cor Biosciences).

### 2.8. Purification of Recombinant Proteins

The supernatants collected at 4–6 days after subculture of established cell line were clarified by centrifugation at 4000 rpm for 15 min and then filtered through a 0.45 µm pore size membrane. The clarified medium was subjected to chromatography purification with Ni-NTA columns. Proteins eluted with gradient of imidazole (200 mM, 300 mM and 500 mM) in elution buffer were concentrated, and buffer was changed into PBS by a PD-10 desalting column (GE 52130800BB). Protein concentration was determined by using a BCA protein assay kit (Biosharp, BL521A, Beijing, China) with bovine serum albumin as the standard. Purified proteins were stored at −80 °C until use.

### 2.9. Immunization and Challenge of Pigs

Twelve ASFV antibody-negative 30-day-old landrace pigs were divided into four groups. There were four pigs in group A. Each pig was intramuscularly immunized with 1 mL emulsion containing 50 μg CD2v protein emulsified with adjuvant MONOTA VG 61 (Seppic). There were three pigs in group B. Each pig in group B was intramuscularly immunized with 1 mL emulsion containing 50 µg CDv and 50 µg p30 proteins emulsified with adjuvant MONOTA VG 61. There were three pigs in group C. Each pig in group C was intramuscularly immunized with 1 mL emulsion containing 50 µg each of CD2v, p30 and K205R proteins emulsified with MONOTA VG 61. The last two pigs were inoculated with PBS as an unimmunized control group D. Twenty-eight days after the first immunization, all pigs were boosted with the same formula and dose as the first immunization. Blood samples were collected on days 0, 14, 28 and 42 for antigen-specific antibody titration. At 42 days post-immunization, all pigs were challenged with 300 HAD_50_ ASFV strain HLJ/18 in 1 mL. The pigs were monitored daily for 21 days post-challenge for clinical signs and mortality. Blood samples from surviving pigs were collected at 3, 6, 7, 15 and 21 days post-challenge for ASFV viremia titration with the HAD assay.

### 2.10. Enzyme Linked Immunosorbent Assay

The antigen-specific antibodies were determined using ELISA. Briefly, 96-well ELISA plates were coated with purified fusion protein in 0.05 M carbonate buffer (pH 9.6) at 4 °C overnight and blocked with 5% skimmed milk for 2 h at 37 °C. After washing three times with PBST (PBS plus 0.5% Tween-20), sera samples were added and incubated for 1 h at 37 °C. Plates were then washed three times with PBST and incubated with horseradish peroxidase (HRP)-conjugated goat anti-mouse IgG for 1 h at 37 °C. Following three washes with PBST, 100 μL of 3,3′,5,5′-tetramethylbenzidine (TMB) was added. The reaction was stopped with 2 M H_2_SO_4_, and the optical density was measured at 450 nm using an ELISA plate reader (Bio-Rad, Hercules, CA, USA).

### 2.11. Hemadsorption Assay

The ASFV was titered by using the hemadsorption (HAD) assay as described previously [3,27]. Briefly, primary porcine PBMCs were seeded in 96-well plates. The 10-fold serially diluted samples were then added to the plates and titrated in triplicate. The quantity of ASFV was determined by identification of characteristic rosette formation representing hemadsorption of erythrocytes around infected cells. HAD was observed for 7 days, and 50% HAD doses (HAD_50_) were calculated by using the method of Reed and Muench.

## 3. Results

### 3.1. Expression, Purification and Characterization of CD2v Protein

**Figure 1:**
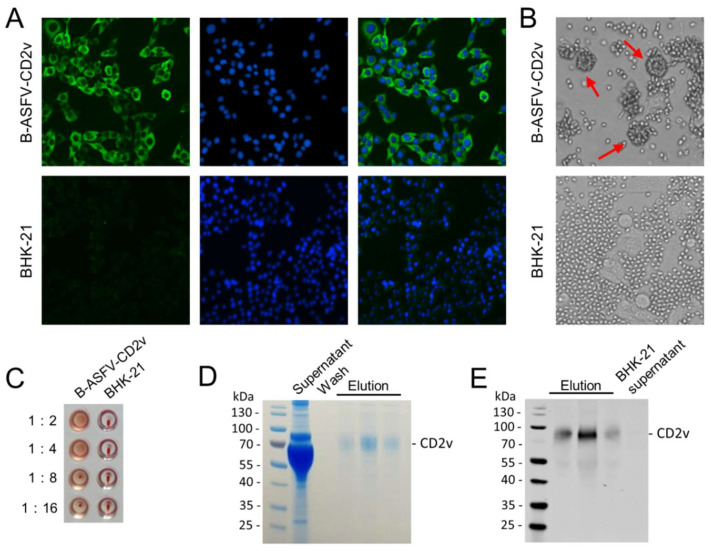
Expression, purification and characterization of CD2v protein of ASFV. (**A**) Identification of recombinant cell line B-ASFV-CD2v by IFA. (**B**) Hemadsorption of CD2v-expressing cell line B-ASFV-CD2v. Specific formations of rosettes are indicated by arrows. (**C**) Hemagglutination of supernatants of B-ASFV-CD2v cell culture. (**D**) SDS-PAGE analysis of purified recombinant CD2v protein. (**E**) Detection of CD2v protein by Western blotting.

After transfection and selection, a cell line stably expressing CD2v protein, designated as B-ASFV-CD2v, was established. The homogeneity of cell line B-ASFV-CD2v was examined by IFA with 6 × His-tag-specific antibody (Figure 1A). The B-ASFV-CD2v cells showed nearly 100% positivity, but the parent BHK-21 cells showed no positive signal. The recombinant cell line has the ability to induce the hemadsorption phenomenon. The CD2v-expressing cells are capable of inducing the rosetting of erythrocytes (Figure 1B). The hemagglutination property of recombinant CD2v was also surveyed in microwell plates. As expected, the clarified supernatants of B-ASFV-CD2v cultures exhibited hemagglutinating activity (Figure 1C). The recombinant CD2v protein was purified from the supernatants of B-ASFV-CD2v cultures by Ni-affinity chromatography. The SDS-PAGE analysis showed that the molecular weight of the recombinant CD2v was about 75 kDa, which was much higher than the expected molecular weight of 26.4 kDa (Figure 1D). The purified CD2v protein was also verified by Western blotting with anti-His-tag antibody (Figure 1E). This result suggested that CD2v was highly glycosylated as previously described. 

### 3.2. Cell-Line-Expressed Protein Antigens Induced Robust Antibody Responses

**Figure 2:**
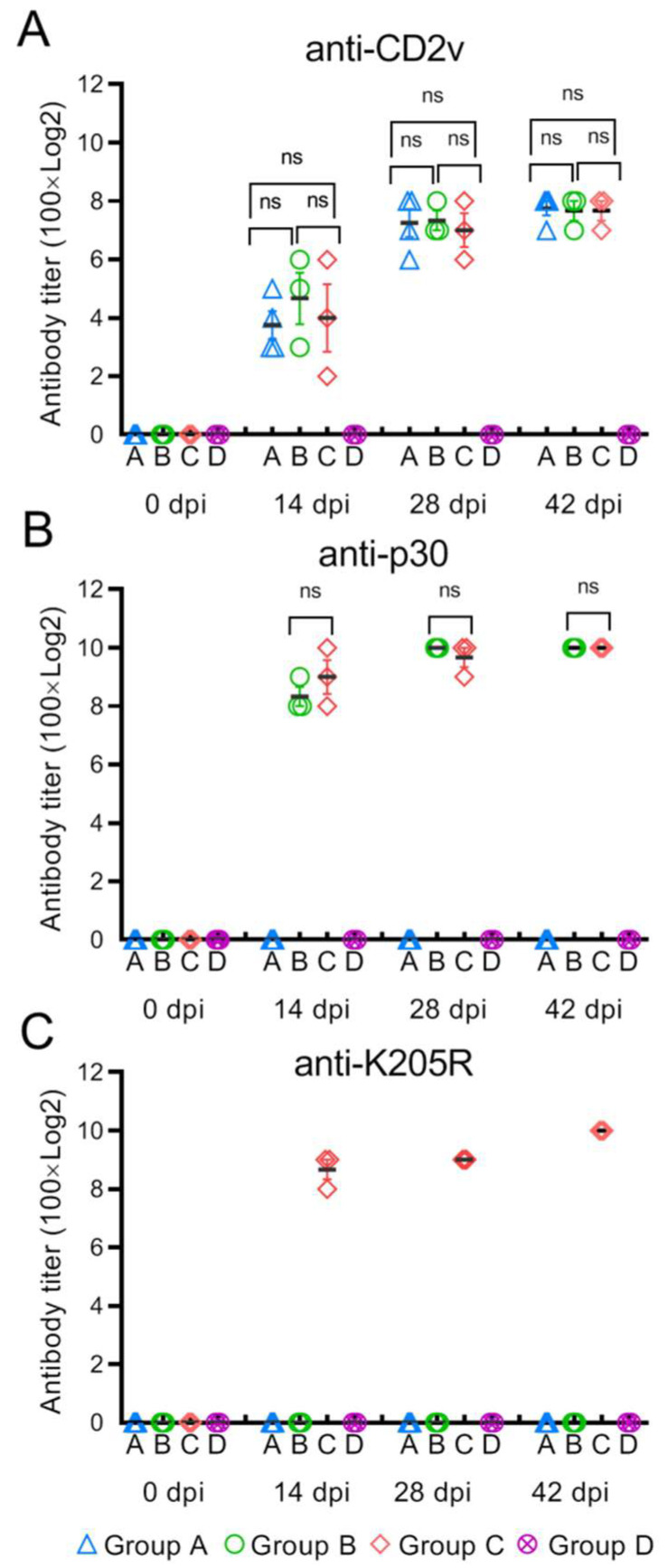
Antibody responses of immunized pigs. Sera samples at different time points were collected. The antibody titers were detected by ELISA with CD2v (**A**), p30 (**B**) and K205R (**C**) protein-coated microplates. The data were analyzed with GraphPad Prism software. Statistical significance was determined by analysis of variance with a multiple-comparison correction (ns, *p* > 0.005).

To survey the immunological properties of CD2v singly or combined with p30 or combined with p30 + K205R, three groups of pigs were immunized with CD2v, CD2v + p30 or CD2v + p30 + K205R. Antigen-specific antibodies in sera from pre- or post-immunization were titrated with ELISA. In all three immunized groups, anti-CD2v antibodies were detected 14 days after immunization. There is no significant difference in the mean antibody titers of groups A, B or C (Figure 2A). The CD2v antibody titers increased at 28 days post-immunization and reached a plateau at 42 days post-immunization (Figure 2A). For the p30 protein immunized in groups B and group C, strong antibody responses were observed at 14 dpi. By day 28 after immunization, antibody titers had reached quite high levels, even comparable to the antibody levels after enhanced immunization. Moreover, there was no significant difference between group B and group C in anti-p30 antibody levels (Figure 2B). In group C, K205R was immunized in combination with CD2v and p30, and K205R also induced strong antibody responses (Figure 2C).

### 3.3. CD2v Provided Partial Immune Protection, but p30 Showed No Synergistic Protective Effect

**Figure 3:**
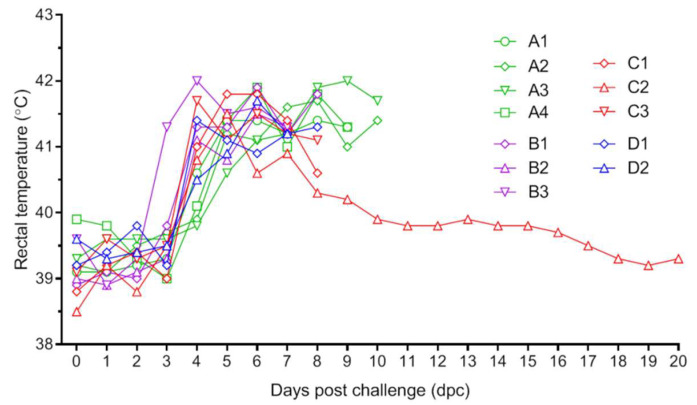
Rectal temperatures of pigs at different days post-challenge. Immunized and control pig were challenged with the virulent ASFV HLJ/18 strain. After challenge, rectal temperatures of each pig were measured daily for 21 days.

To evaluate the immune protection of three formulations of the vaccine, all pigs were challenged with ASFV strain HLJ/18 at 42 days post-immunization. Two unimmunized pigs in group D showed ASFV clinical manifestations from day 4 post-challenge and died on days 7 and 8. The average time for group A to start showing clinical symptoms was 4.8 days, 0.8 days later than the control group. The average survival time was 9.5 days, which was 2 days longer than that of the control group (Figure 3). The CD2v immunization delayed the onset of viremia, and all four pigs in group A showed negative viremia on day 3 post-challenge, while the levels of viremia on day 6 were similar to those of the control group (Table 1). These results preliminarily demonstrate that CD2v could only provide partial immune protection against the virulent strain HLJ/18 in the early infection stage.

In group B, the average time of onset of clinical symptoms was 3.7 days, which was near that of the control group. All three pigs died on day 7 or 8 post-challenge with an average survival time of 7.3 days, which was close to the control group (Figure 3). On day 3 post-challenge, all three pigs developed viremia (Table 1). 

In group C, all three pigs developed fever and showed clinical symptoms of ASFV on day 4 post-challenge. Two of the three pigs died on day 8. Unexpectedly, pig C2 survived throughout the 21-day observation period, and its body temperature dropped to normal on day 15 post-challenge (Figure 3). The level of viremia in the surviving C3 pig also decreased and was found to be negative on day 21 at the end of the observation period (Table 1).

## 4. Discussion

The CD2v protein is so far the only experimentally identified outer membrane glycoprotein of ASFV [28]. The protein binds to pig erythrocytes and mediates invasion and infection of the virus. The CD2v gene is not an essential gene for virus replication, but this gene is one of the virulence-determining genes. Deletion of CD2v gene attenuated the virulent BA71 strain [29] but slightly attenuated the HLJ/18 strain [27] or did not attenuate the Georgia/2010 strain [30]. These findings suggest that the molecular basis of the virulence of ASFV may vary among strains, and the immune characteristics of CD2v protein may also vary among strains. Previous studies have shown that CD2v protein could induce virus-neutralizing antibodies and provide protection against homologous virulent strains. Strain HLJ/18, a representative strain of the genotype II strains that emerged in China in 2018, was highly virulent. Whether the expressed CD2v protein could provide immune protection against HLJ/18 was unknown. This information was essential for the development of ASFV subunit vaccines. In this study, to ensure that the expressed CD2v protein was well post-translational modified, we adopted a mammalian cell line expression system to express the CD2v protein. The results showed that the molecular weight of the CD2v protein expressed by the cell line was higher than predicted. The purified CD2v protein appears as a smear on SDS-PAGE. These results altogether indicated that the cell-line-expressed CD2v protein was fully glycosylated. The CD2v-expressing cells demonstrated hemadsorption characteristics, and the purified CD2v protein also showed hemagglutination properties. These results all match the characteristics of the CD2v protein that have been reported.

After immunization in pigs, CD2v protein could induce specific antibody responses. Although the immunized pigs were not completely protected against the virulent virus challenge, all four pigs developed disease and died. However, the average manifestation onset time in group A was about one day later than that in the control group. CD2v immunization also delayed the onset of viremia and prolonged the survival time of the immunized pigs. These results suggest that CD2v protein immunization provided only partial protection at the early stage of infection. Obviously, more immune protective antigens are needed to act synergistically with CD2v to improve the protective effect of the ASFV subunit vaccine.

p30 is another antigenic protein that has been reported to confer immune protection [12,14]. Expecting to improve immune protection, we combined CD2v with p30 protein for immunization. In this group, strong antibody responses to p30 and CD2v were induced in all immunized pigs, and there was no significant difference in anti-CD2v antibody titers between groups immunized with CD2v alone and in combination with p30. This result indicates that p30 protein did not interfere with CD2v antibody response when immunized in combination. However, we did not observe a synergistic protective effect of p30 protein on CD2v protein. On the contrary, no significant difference was observed in the manifestation onset time, viremia level at 3 dpc or survival time between group B and the control group D. These results seem to suggest that p30 protein may play an inhibitory role in the immune protective properties of CD2v protein. Of course, further experiments are needed to verify this hypothesis.

K205R was a highly immunogenic protein of ASFV [26], but the role of this protein in immune protection was unknown. To observe the immune effect of K205R protein, we immunized a group with a combination antigen of CD2v, p30 and K205R. In this group, strong antibody responses were all induced. There were no interference effects between any of the three antigens. The results of group B seem to indicate that p30 protein has an inhibitory effect on the immune protection of CD2v. However, the viremia titers on the third day after the challenge in group C suggested that the immune protection at the early stage of infection seemed to be restored. It is interesting that one pig out of three in group C was completely recovered. The pig survived throughout the observation period, with a drop in rectal temperature to normal level, disappearance of symptoms and clearance of viremia. The humoral immune mechanisms responsible for this protection are not conclusive. Since CD2v or combinate could only provide partial protection against current circulating strain HLJ/18, more protective antigens need to be identified for the development of effective ASFV subunit vaccines. It is also necessary to investigate the synergistic or antagonistic effects between antigens.

## Figures and Tables

**Table 1:** Viremia titers of pigs at different time points post-challenge.

Group	Pig No.	Viremia Titers * (Log HAD_50_/mL)				
		3 dpc	6 dpc	7 dpc	15 dpc	21 dpc
A	A1	<1.7	7.2	6.95	/	/
	A2	<1.7	6.45	6.45	/	/
	A3	<1.7	5.45	5.45	/	/
	A4	<1.7	6.1	6.3	/	/
B	B1	5.7	7.1	/	/	/
	B2	2.3	6.54	/	/	/
	B3	3.7	5.45	/	/	/
C	C1	<1.7	5.7	/	/	/
	C2	<1.7	4.2	4.2	3.45	<1.7
	C3	2.3	6.95	/	/	/
D	D1	5.2	7.1	7.2	/	/
	D2	6.27	7.45	/	/	/

* The titers of samples with negative HAD result at initial dilution of 1:50 are expressed as <1.7/not tested or unavailable for animals that died.

## Data Availability

The authors declare that the data supporting the findings of this study are available within the article or upon request to the corresponding authors.
